# Opportunity of Next-Generation Sequencing-Based Short Tandem Repeat System for Tumor Source Identification

**DOI:** 10.3389/fonc.2022.800028

**Published:** 2022-02-11

**Authors:** Anqi Chen, Lei Xiong, Yiling Qu, Shihan Xi, Ruiyang Tao, Chengtao Li, Suhua Zhang

**Affiliations:** ^1^ Shanghai Key Laboratory of Forensic Medicine, Shanghai Forensic Service Platform, Academy of Forensic Science, Ministry of Justice, Shanghai, China; ^2^ Department of Forensic Medicine, School of Basic Medical Sciences, Shanghai Medical College, Fudan University, Shanghai, China

**Keywords:** forensic identification methods, short tandem repeat (STR), next-generation sequencing (NGS), tumor source identification, forensic genetics

## Abstract

Personal identification using the tumor DNA not only plays an important role in postoperative tissue management but also might be the only accessible source of biological material in forensic identification. Short tandem repeat (STR) is the worldwide accepted forensic marker; however, widespread loss of heterozygosity (L) in tumor tissues challenges the personal identification using the conventional capillary electrophoresis (CE)-based STR typing system (CE-STR). Because the tumors are mixtures of tumor cells and basal cells, we inferred that every germline-originated allele should be detected if the detection method was sensitive enough. Next-generation sequencing (NGS) is known as a highly sensitive application, which might be a promising tool for tumor source identification. In the study, we genotyped and compared the STR results between the platforms, and we found that the concordance was only 91.43%. Higher sensitivity did help identify more germline-originated alleles as expected, and 93.89% of them could be captured by using an NGS-based STR system (NGS-STR). The identity-by-state (IBS) scoring system was applied to generate a new tumor source identification method based on NGS-STR, and the number of loci with 2 identical alleles (A_2_) proved to be an ideal criterion for the larger area under the receiver operating characteristic (ROC) curve (AUC). Both the sensitivity and specificity were above 98% in the cutoff of A_2_ to distinguish the paired carcinoma (PC) sample group from the unrelated individual (UI) group, the simulated full sibling (FS) group, and the simulated parent–offspring (PO) group.

## Introduction

In the previous study, we have evaluated the short tandem repeat (STR) status in the paired tumor tissues and found that the mutations are widespread across the tumor types (1). DNA profiling using the STRs for personal identification has been accepted in forensic applications worldwide (2, 3). The STR instabilities in tumors challenge the reliable interpretation of the genetic profiles and further interfere with the individual identification analyses and paternity cases (4). Therefore, the tumor tissues are not accepted as a routine biological material in forensic medicine. However, it is also possible for the tumor tissue to become a biological sample in the case of either tumor source identification or paternity testing. Although an alternative method using the genetic markers with lower mutation rates (e.g. SNPs) has been established (5), there remains an opportunity for the traditional STR markers since nearly all of the countries have established DNA databases based on the STR profiles (6). To maintain close contact with the databases, it is necessary to generate the method for tumor source identification using the STR markers.

Published studies (4, 7) classify the STR status in tumors into five categories, which includes stable (S), complete allelic loss of heterozygosity (L), partial allelic loss of heterozygosity (pLOH), occurrence of an additional allele (Aadd), and occurrence of a new allele (Anew) using the capillary electrophoresis (CE)-based STR genotyping (CE-STR). Tumor tissues are the mixtures of basal cells and tumor cells, so the DNA profiles should possess the characteristics of both cell types. Obviously, Anew and L might be mistaken conceptions. In real clinical practice, the tumor contents of the tissues are above 30% (8), whereas the detection of the minor donor using CE platforms is only about 5-fold excess (20% of the total STR profile) of the major donor (9). Therefore, signals from the minor cells (whose content was below 20%) might be masked at high mixture ratios and subsequently resulted in the observation of L and Anew. Based on the composition of tumor tissues, we suspected that all alleles from the stromal cells (which are known as germline-originated alleles) should be detected in the tumor samples if the detection platform was sensitive enough. The high resolution over high sensitivity of the next-generation sequencing (NGS) has led to the resolution of many scientific issues (10). To date, there has been no research on tumor source identification using the NGS-based STR typing system (NGS-STR). Since the mixture interpretation has been challenged due to the low sensitivity level of CE-STR, could the NGS-STR seize the opportunity to overcome the limitations?

In order to investigate if all germline-originated alleles could be detected using the NGS-STR, all samples had been typed by both the conventional CE-STR systems and a worldwide validated NGS-STR platform. At the same time, a new NGS-STR-based tumor source identification method has also been generated. It was the first attempt to solve the tumor source identification problem using the NGS-STR, which was valuable not only to fill the gaps in knowledge of forensic science but also to provide an alternative method for tumor source identification.

## Materials and Methods

### Sample Collection and Preparation

Fifty-five paired tumor samples and the peripheral blood samples from seventy-five unrelated individuals (UIs) were collected for the study. The paired tumor samples consisted of 6 gastric cancer cases, 33 colorectal cancer cases, 2 breast cancer cases, 4 pancreatic cancer cases, 2 lung cancer cases, 2 esophageal cancer cases, 2 renal cell cancer cases, and 4 hepatocellular cancer cases. The patients underwent surgical tumor resection at the Changhai Hospital, Second Military Medical University, Shanghai, and Huadong Hospital Affiliated with Fudan University, Shanghai, in 2013–2019. All samples were recruited upon the approval of the Ethics Committee of the Academy of Forensic Science, Ministry of Justice, China. Written informed consent was provided by each participant (No. SJY2013-W002, approved January 4, 2013). The relative percentage of tumor cells to nucleated cells of the tissue samples was assessed by a senior pathologist after H&E staining. Samples with at least 30% tumor cells were considered for further study. Peripheral blood cells or para-carcinoma tissues were used for control DNA isolation.

Tumor DNA was extracted using a DNeasy Blood & Tissue Kit (Qiagen, Valencia, CA, USA). The DNA from the blood controls and the UIs was extracted from 100 μl of peripheral blood using a QIAamp DNA Blood Kit (Qiagen, Venlo, The Netherlands). All DNA was extracted in accordance with the manufacturer’s recommendations and quantified using a Qubit fluorometer (Life Technologies, Carlsbad, CA, USA). All extracted DNA was stored at –80°C until use.

### Evaluation of Short Tandem Repeat Mutation Status Using the Capillary Electrophoresis-Based Systems

The STR status was determined with either the Goldeneye^®^20A Forensic Identifier Kit (PeopleSpot, Beijing, China) or the SiFaSTR™ 23-plex system. Fluorescent multiplex PCR was performed in accordance with the manufacturer’s instructions. Genotyping was performed in a 3100 ABI Prism Genetic Analyzer (Applied Biosystems, Foster City, CA, USA) by using GeneMapper Software (Applied Biosystems, USA). A detection threshold of 50 relative fluorescence units (RFU) was used for the analysis of the sample profiles, and the results were reviewed by two experienced technicians. The genotypes of the somatic STRs were detected in all paired samples, and the STR status was evaluated against the control STR type. In total, four types of mutations were classified for the respective samples, namely, L, pLOH, Aadd, and Anew. Stable (S) refers to the samples with stable STRs (11).

### Library Preparation, Sequencing, and Data Analysis

Genomic DNA of 1 ng was used for the test. Libraries were constructed using the ForenSeq™ DNA Signature Prep Kit (Illumina, CA, USA) following the manufacturer’s instructions. The NGSs were performed on the Miseq FGx™ sequencer (Illumina, CA, USA). The analysis of the sequencing data was performed using the built-in ForenSeq™ Universal Analysis Software (UAS, Verogen, San Diego, CA, USA) with its default settings. The reports given by UAS were provided with allele names, genotypes, and the corresponding read coverages. The default thresholds for each of the loci could be found in **Supplementary Material 1**, and the sequence data could be obtained from **Supplementary Material 2**. Depending on whether the allele was typed or not, the report could be downloaded by either Sample Summary Report (the results were given using the built-in IT) or Sample Details Report (the results were given using the built-in IT). In the present study, the STR profiles for the normal samples were genotyped using the built-in IT. The STR profiles for the tumor samples were compared using both thresholds, namely, NGS-IT and NGS analytical threshold (NGS-AT).

### Statistical Analysis

The number of matched STR locus with 0 identical allele (A_0_), 1 identical allele (A_1_), and 2 identical alleles (A_2_), as well as the identity-by-state (IBS) scores, were assessed within the paired carcinoma (PC) sample group, the tumor-UI group, the tumor-simulated full sibling (FS) group, and the simulated tumor-simulated parent–offspring (PO) group. The genotype data of FS pairs and PO pairs were generated by simulation. In short, we first simulated the PO pairs by randomly assigning a breeding patch to each individual of the first generation based on the data of the normal sample. For each of the FS pairs, we again assigned a breeding patch to each individual at random. All simulated pedigrees were manually checked, which are consistent with Mendel’s laws. Statistical analyses were performed using Prism 4.0 software (GraphPad, San Diego, CA, USA). The statistical analyses were performed using Student’s t-test, and the differences were considered significant if the p < 0.05.

## Results

### Distribution of the Short Tandem Repeat Mutations in Two Different Platforms

**Figure 1:**
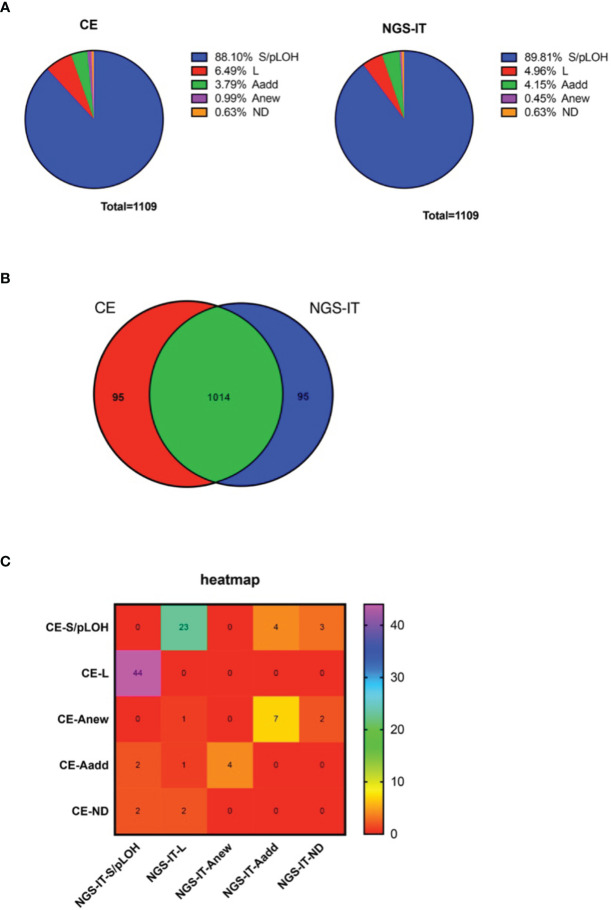
The classification of STR status with the CE and NGS platforms. **(A)** Distribution of the STR status resulted from two platforms. Left, CE; right, NGS-IT. **(B)** Venn diagram illustrating the overlap between the STR status that resulted from different platforms. **(C)** Heat map depicts the STR status between CE and NGS-IT. The rainbow color key represents the number of the loci. Purple color represents an increasing number of the loci, and red color indicates decreasing number of the loci. STR, short tandem repeat; CE, capillary electrophoresis; NGS, next-generation sequencing; NGS-IT, NGS interpretation threshold.

The tumor samples were collected from our previous study (1, 11). To investigate the influence the mutations played on the NGS platform, the tumor samples that harbored at least one STR mutation were preferred. In total, there were fifty-five paired tumor samples collected in the study. The comparison was made using the 1,109 autosomal STR loci shared by both platforms. The result of the STR status showed that most of the loci were remarked as S/pLOH, and the percentage from the NGS-IT (89.81%) was higher than that exhibited by CE (88.10%). L was the second most frequently occurring mutation in the study (CE, 6.49%; NGS-IT, 4.96%), followed by Aadd (CE, 3.79%; NGS-IT, 4.15%) and Anew (CE, 0.99%; NGS-IT, 0.45%). Approximately 0.6% of the loci failed to be typed by CE, and the percentage in NGS-IT was the same (**Figure 1A**). The comparison in genotype concordance was summarized in **Figure 1B**. Although 91.43% (1014/1109) of the genotyping result was consistent, there were 95 loci that remained different between the two platforms (**Figure 1B**). The detailed STR status of the 95 dis-concordant typing results is summarized in **Figure 1C**. Most of the conflicts focused on the definition of L and S/pLOH, followed by Aadd and Anew. A total of forty-four CE-interpreted L were defined as S/pLOH by NGS-IT, and twenty-three NGS-IT-defined L were interpreted as S/pLOH by CE. Seven CE-defined Anew loci were marked as Aadd by NGS-IT. In summary, the NGS-IT resulted in more allele S/pLOH and less Anew than CE; meanwhile, CE produced more L and Anew than NGS-IT.

### Nearly All of the Germline-Originated Alleles Were Found in Tumor Samples Using Next-Generation Sequencing Analytical Threshold

**Figure 2:**
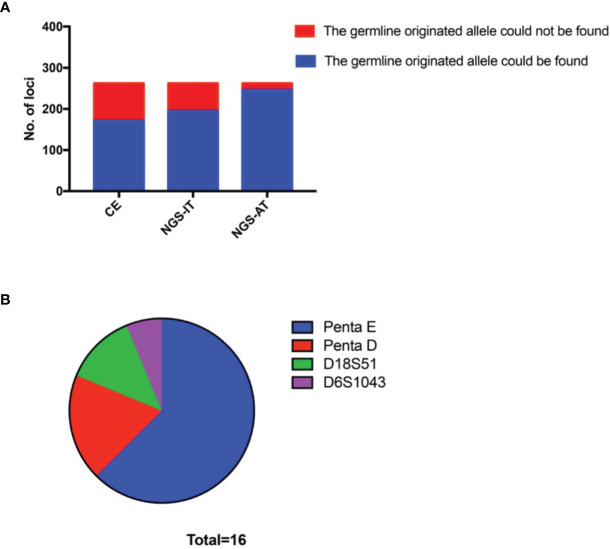
More germline-originated alleles could be found by using an NGS-based platform. **(A)** Number of the germline-originated allele being detected in tumor samples by CE, NGS-IT, and NGS-AT. **(B)** Distribution of the loci whose germline-originated allele could not be found by NGS-AT. NGS, next-generation sequencing; CE, capillary electrophoresis; NGS-IT, NGS interpretation threshold; NGS-AT, NGS analytical threshold.

Based on the theory of tumor mixtures, we supposed that all germline-originated alleles could be found in the sensitivity that was good enough. Based on the varied cell proportions, the typing results showed either imbalanced genotyping or dropout for the low coverage. Apparently, the NGS was likely to screen out more germline-originated signals. To find the alleles with low signals, we read the results using the AT (which typed the allele using the lower reads). There were 262 STR mutations detected by CE-based platforms in total (**Figure 2A**). More germline-originated alleles were found by the NGS-based platform (74.43%), and nearly 93.89% (246/262) of them could be detected under the NGS-AT (**Figure 2A**). The remaining sixteen loci, whose alleles failed to be found, showed locus-specific characteristics. Although the STR mutations occurred in all of the STR loci (data not shown), the sixteen loci were only limited to Penta E, Penta D, D18S51, and D6S1043. A large proportion of the mutations were observed in Penta E (**Figure 2B**).

### Tumor Source Identification Using the Identity-by-State Scoring System

**Figure 3:**
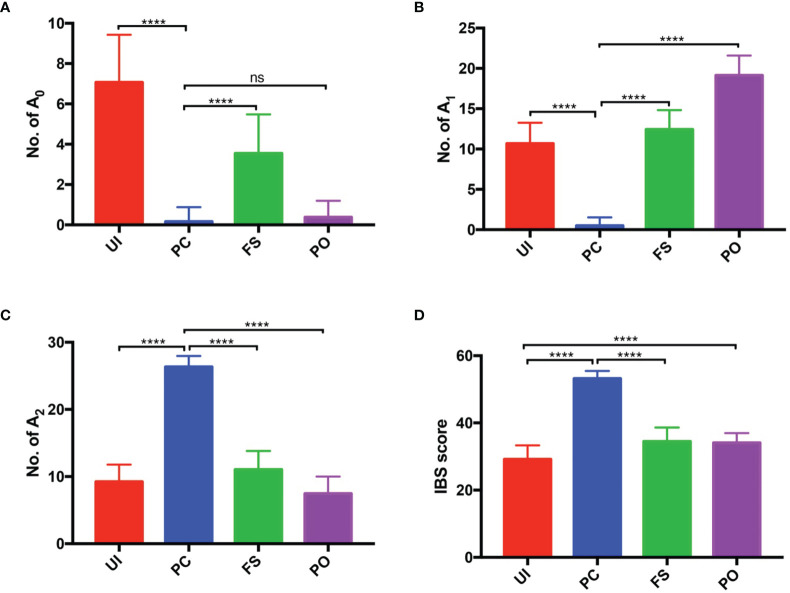
Histogram of number of A_n_ in PC, UI, and FS. **(A)** Number of the loci with 0 identical alleles (A_0_). **(B)** Number of the loci with 1 identical allele (A_1_). **(C)** Number of the loci with 2 identical alleles (A_2_). **(D)** IBS score. PC, paired carcinoma; UI, unrelated individual; FS, full sibling; IBS, identity by state. ns, no significance; ****P < 0.0001.

**Figure 4:**
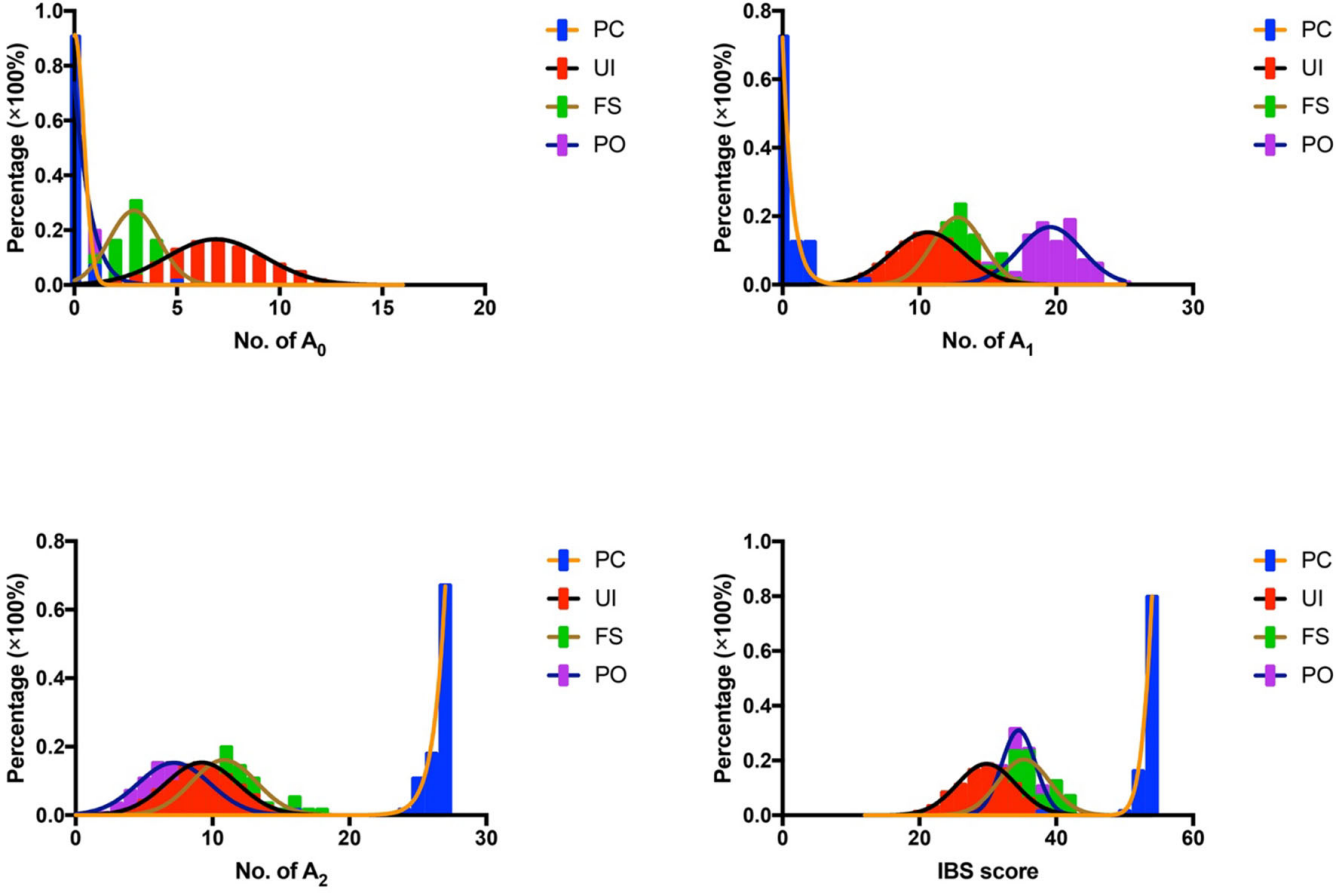
Probability distribution of A_0_, A_1_, A_2,_ and IBS in UI, FS, and PO. IBS, identity by state; UI, unrelated individual; FS, full sibling; PO, parent–offspring.

In the study, we adopted the IBS scoring system for tumor source identification. Since the tumor tissue consisted of tumor cells and basal cells, each of the germline-originated alleles should be detected in the tumor samples. Compared to the UI, FS, and PO, more identical alleles should be found in PC. As shown in **Figure 3**, significant differences had been detected except for A_0_ values in PO. In addition, the probability distributions of A_0_, A_1_, A_2_, and IBS in the UI, PC, FS, and PO were also analyzed (**Figure 4**). According to the A_0_ distributions in each group, there is a probability of two tissues being PC with a lower A_0_ value (**Figure 4**). As for the A_1_, A_2_, and IBS score distributions, the probability of the two tissues being PC increased with an increasing number of values.

**Figure 5:**
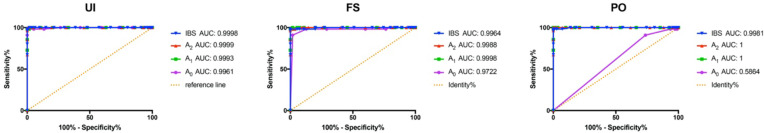
ROC curves of the four values (A_0_, A_1_, A_2_, and IBS score) in UI, FS, and PO. ROC, receiver operating characteristic; IBS, identity by state; UI, unrelated individual; FS, full sibling; PO, parent–offspring.

Given that all the values (A_0_, A_1_, A_2_, and IBS score) led to a significant difference among the groups (**Figure 3**), we tried to find the most suitable criteria prior to performing forensic discriminations. The area under the receiver operating characteristic (ROC) (AUC) for A_0_ was only 0.5864 in PO, which demonstrated the lowest discriminating power. AUC values for A_2_ were the highest in UI and PO, and the discriminating power of A_1_ was the largest in FS. Considering the comparative values between A_1_ and A_2_, we adopted A_2_ as the criteria to perform the following discriminating test for convenience (**Figure 5**).

We estimated both the sensitivity and specificity of A_2_ using the non-linear fitting function. In view of the data listed in **Table 1**, Youden’s index reached its peak (99.39%) at the cutoff of 16 in UI. The largest values in FS and PO were 98.18% and 99.09%, respectively; at this point, the corresponding thresholds were 19 and 14. The sensitivity and specificity to distinguish PC and UI exceed 95% when the A_2_ was set in the range of 14 to 25. To achieve the same distinguishing power to identify PC to FS and PO, the threshold should be set in the range of 18~25 and 13~22, respectively (**Table 1**).

**Table 1:** Sensitivity, specificity, and Youden’s index at various cutoff points of A_2_.

Cutoff	UI			FS			PO		
	Sensitivity%	Specificity%	Youden’s index	Sensitivity%	Specificity%	Youden’s index	Sensitivity%	Specificity%	Youden’s index
≥1	100	0.04848	0.04848	100	3.636	3.636	100	0.9091	0.9091
≥2	100	0.04848	0.04848	100	3.636	3.636	100	0.9091	0.9091
≥3	100	0.1697	0.1697	100	3.636	3.636	100	0.9091	0.9091
≥4	100	0.7515	0.7515	100	3.636	3.636	100	4.545	4.545
≥5	100	2.642	2.642	100	3.636	3.636	100	11.82	11.82
≥6	100	7.03	7.03	100	3.636	3.636	100	22.73	22.73
≥7	100	14.47	14.47	100	3.636	3.636	100	38.18	38.18
≥8	100	24.46	24.46	100	7.273	7.273	100	53.64	53.64
≥9	100	39.25	39.25	100	18.18	18.18	100	67.27	67.27
≥10	100	54.59	54.59	100	29.09	29.09	100	79.09	79.09
≥11	100	68.78	68.78	100	40	40	100	85.45	85.45
≥12	100	80.65	80.65	100	60	60	100	92.73	92.73
≥13	100	89.55	89.55	100	74.55	74.55	100	97.27	97.27
≥14	100	95.56	95.56	100	85.45	85.45	100	99.09	99.09
≥15	100	98.3	98.3	100	89.09	89.09	100	100	100
≥16	100	99.39	99.39	100	90.91	90.91	100	100	100
≥17	98.18	99.85	98.03	98.18	96.36	94.54	98.18	100	98.18
≥18	98.18	99.95	98.13	98.18	98.18	96.36	98.18	100	98.18
≥19	98.18	100	98.18	98.18	100	98.18	98.18	100	98.18
≥20	98.18	100	98.18	98.18	100	98.18	98.18	100	98.18
≥21	98.18	100	98.18	98.18	100	98.18	98.18	100	98.18
≥22	96.36	100	96.36	98.18	100	98.18	96.36	100	96.36
≥23	96.36	100	96.36	96.36	100	96.36	85.45	100	85.45
≥24	96.36	100	96.36	96.36	100	96.36	85.45	100	85.45
≥25	96.36	100	96.36	96.36	100	96.36	85.45	100	85.45
≥26	85.45	100	85.45	85.45	100	85.45	85.45	100	85.45
≥27	67.27	100	67.27	67.27	100	67.27	67.27	100	67.27

UI, unrelated individual; FS, full sibling; PO, parent–offspring.

## Discussion

In the previous study, we have successfully employed the STR markers to evaluate the tumor hypermutability and found widespread alterations across the tumor types (1, 11). Tumors grow with the accumulation of mutations, and they are not preferred as the biological sample. However, the tumor tissue is inevitable to become a prime research object in the service of tumor source identification. In addition, the archived tumor tissue might be the only accessible biological sample of a person in the world, which made the typing method for tumor tissue much more important.

The STR mutations have limited the usage in tumor source identification. Up to now, no guidelines have been released for tumor source identification. To avoid the mutations that occurred in the genetic markers, Sun et al. (5) have established the single-nucleotide polymorphisms (SNPs) as the biomarkers to identify the source of tumor tissues. SNP markers are known for their low mutation rate and small amplicon size, which might reduce ambiguous typing results. However, the SNP markers are not as popular as the conventional STR markers. STR databases remain the most fast-growing and popular forensic databases (12, 13). Therefore, generating a method for tumor identification using the STR markers would be much more valuable than any other genetic marker.

Conventional STR typing is produced by CE-based typing systems, which have sorted the STR mutations into four categories (11, 14, 15). Zhao et al. (15) once generated a method for tumor source identification based on Fisher’s discriminant functions, but the model was constructed using only 15 STRs. With the update of chemical protocols, more and more STRs have become indispensable markers for forensic identification (16). The cost of genome sequencing has plunged in recent years, which promotes the development of large panels. Nowadays, large panels have gained a majority of global markets for the NGS tests (17, 18). To the best of our knowledge, no solution has been carried out for tumor source identification using STR markers by NGS so far. With the mixtures of tumor cells and basal cells, the DNA profiles of tumor samples should show the profiles of both the tumor cells and the basal cells. Therefore, the conventional description of L and Anew could be misinterpretations in theory. The CE-based typing platforms possess inferior limited detection, which is doomed to lose many expected germline-originated signals. How could we make the right conclusions if we did not recognize the status correctly? The highly sensitive NGS methods have helped solve many scientific problems (19); after that, more and more customized and commercial panels have been developed for forensic application (20). Verogen’s ForenSeq™ DNA Signature Prep Kit is the most popular commercial kit in forensic laboratories, and the performances have been well validated by the researchers both at home and abroad (21, 22). The advantage of high sensitivity suggests that the kit might be a promising tool for identifying the tumor samples. Based on all of the above, we used the ForenSeq™ DNA Signature Prep Kit to investigate the real STR status of the tumor tissues.

In the study, we collected fifty-five paired tumor samples that harbored at least one STR mutation. All paired samples were sequenced using Verogen’s ForenSeq™ DNA Signature Prep Kit, and comparable STR mutation landscapes were achieved between the two platforms. We observed that L was the most frequently occurring mutation, followed by Aadd and Anew (**Figure 1A**). Fewer loci typed with L and Anew by NGS-IT indicated that the NGS has better sensitivity. As mentioned above, we speculated that the Anew and L were not the real STR status. They should be Aadd and pLOH by restoring the germline-originated allele in theory. As shown in **Figure 1A**, more S/pLOH status and less L status were detected by NGS-IT. Meanwhile, the frequency of Anew was smaller than that of CE. The result suggested that the high sensitivity of NGS did help recognize the real genetic status of the tumor samples. To further investigate the difference, we summarized the genotype concordance between the platforms and found that 95 loci were being typed differently (**Figure 1B**). Furthermore, we observed that the conflicts mainly gathered around the interpretation of L vs. S/pLOH and Aadd vs. Anew (**Figure 1C**). The occurrences of L and Anew might result from the allelic loss, which was in concordant with our hypothesis. The results suggested that sensitivity played an important role in the interpretation of L and Anew. The ForenSeq™ DNA Signature Prep Kit is designed for single-source DNA, so the imbalanced or low-coverage alleles are likely to be flagged and un-typed by the IT. Tumor tissues are the mixtures with unknown cell proportions; therefore, the typing results probably reflect the imbalance of cell proportions. Consequently, the tumor signals are likely to be un-typed with the NGS-IT. For the UAS, the AT of lower reads was applied in the study, and it might provide more allele information than the NGS-IT. To explore if all germline-originated alleles could be found in tumor samples, we checked the data of all L, pLOH, and Anew using the NGS-AT. As expected, more potential germline-originated alleles had been found (**Figure 2A**), which suggested that NGS-AT might be more suitable for tumor genotyping. The STR mutations were widespread across the loci (data not shown), and all germline-originated alleles had been found except for Penta E, Penta D, D18S51, and D6S1043 (**Figure 2B**). Therefore, we suspected that it might be the low coverage that caused the allelic loss, and the relationship between the coverage and allele calling remained to be discovered further. In general, the results indicated that the four loci might not be suitable for tumor identification in the current situation for the trend of missing allele.

The study began with the hypothesis that every germline-originated allele could be found in tumor tissues. The results that we obtained so far basically supported the hypothesis, and now it was time to establish a method for tumor source identification. As we all know, polymorphism is basic to forensic genetics (23), and different people possess a different combination of STRs. The samples in the PC group were likely to share more identical alleles than the UIs in theory, and larger A_2_ values were detected in PC than in UI, FS, and PO, as expected. The differences are the basis for discrimination. The A_n_ distributions were conformed with expectations because significant differences (p < 0.0001) had been observed (**Figures 3** and **4**). It is known that the AUC summarized the discriminative ability of a test across the full range of cutoffs, and it reflects how good the test is at distinguishing the positive and negative groups. In general, the greater the AUC, the better the test (24). In order to screen out the best value for the further discrimination test, we analyzed the AUC value under the A_0_, A_1_, A_2,_ and IBS scores. The ROC curves showed that A_2_ possessed a larger AUC value, which made it a candidate for cutoff prediction (**Figure 4**). The sensitivity and specificity of a test largely depend on the level that has been chosen as the cutoff point for positive or negative (24), and Youden’s index is known as a single statistic that captures the performance of a dichotomous test (25). Based on the data, Youden’s index reached 95% when the thresholds of A_2_ were set in the range of 14~25, 18~25, and 13~22, in the UI, FS, and PO, respectively. The results implied that 1) the samples might not come from a UI if A_2_ ≥ 13; 2) the tumor might come from the patient himself if A_2_ ≥ 18.

The significance of the research is more than a method construction, and the high accuracy in tumor source identification using the NGS-based STR typing might shed light on the development of clinically relevant NGS panels. The rapid developments of precision medicine have launched a new era in medicine (26). The reducing cost in NGS makes the applications of larger panels more acceptable in the clinic. The targeted NGS-based multigene testing panels always provide comprehensive analysis, which has played an important role in clinical decision making (27). The autosomal STRs are the globally recognized markers for forensic identification (2), and the advantage could be further developed during cancer therapy. The hospitals might face the charges of misdiagnosis that results from the improper management of postoperative tissue. Careless techniques might muddle up the samples during the test. Likewise, the insurance companies might be troubled when patients provide false tumor tissue information for insurance claims. Since more and more pathological tumor tissues would be sequenced for the purpose of targeted therapy, why not mix the biological identification markers in the customized panels just in case?

## Conclusion

In summary, this is the first research on tumor source identification using the NGS-STR typing, which filled up the blanks in this field. Besides, the method was advantageous in spreading and application because it was established based on a popular commercial kit. Moreover, the feasibility of tumor source identification using the NGS-STR might shed light on the new trend of NGS panel development in clinical practice.

## Data Availability Statement

The datasets presented in this study can be found in online repositories. The names of the repository/repositories and accession number(s) can be found below: NCBI with accession PRJNA793278 link: https://www.ncbi.nlm.nih.gov/sra/?term=PRJNA793278.

## Ethics Statement

The studies involving human participants were reviewed and approved by the Ethics Committee of the Academy of Forensic Science, Ministry of Justice, China. The patients/participants provided their written informed consent to participate in this study.

## Author Contributions

Conceptualization: CL and SZ. Methodology: CL and SZ. Formal analysis: AC. Investigation: AC. Resources: CL and SZ. Data curation: AC, LX, YQ, and SX. Writing—original draft preparation: AC. Writing—review and editing: AC, RT, CL, and SZ. Visualization: AC. Supervision: CL and SZ. Project administration: CL and SZ. Funding acquisition: CL and SZ. All authors listed have made a substantial, direct, and intellectual contribution to the work and approved it for publication.

## Funding

This study was supported by grants from the National Youth Top-notch Talent of Ten Thousand Program WRQB2019, the Youth Science and technology innovation leader of Ten Thousand Program 2018RA2102, and the National Natural Science Fund of China (81930056). The funders had no role in study design, data analysis, publishing decisions, or manuscript preparation.

## Conflict of Interest

The authors declare that the research was conducted in the absence of any commercial or financial relationships that could be construed as a potential conflict of interest.

## Publisher’s Note

All claims expressed in this article are solely those of the authors and do not necessarily represent those of their affiliated organizations, or those of the publisher, the editors and the reviewers. Any product that may be evaluated in this article, or claim that may be made by its manufacturer, is not guaranteed or endorsed by the publisher.
